# The correlation between follicular fluid pregnancy-associated plasma protein A levels, fertilization, and embryo quality in GnRH agonist and GnRH antagonist protocols in ART cycles

**Published:** 2012-09

**Authors:** Razieh Dehghani Firouzabadi, Farnaz Mohammadian, Mehri Mashayekhy, Robab Davar, Maryam Eftekhar

**Affiliations:** 1 *Department of Obstetrics and Gynecology, Research and Clinical Center for Infertility, Shahid Sadoughi University of Medical Sciences, Yazd, Iran.*; 2 *Department of Obstetrics and Gynecology, Zanjan University of Medical Sciences, Zanjan, Iran. *; 3 *Department of Obstetrics and Gynecology, Arak University of Medical Sciences, Arak, Iran. *

**Keywords:** *Pregnancy-associated plasma protein A*, *Follicular fluid*, *GnRH agonist*, *GnRH antagonist*, *Assisted reproductive technology*

## Abstract

**Background: **Determination of oocyte fertilization and embryo quality are one of the most important purposes in ART cycles. Follicular fluid provides an important microenvironment for development of oocytes and some biochemical characteristics of the follicular fluid, such as pregnancy-associated plasma protein-A (PAPP-A), may play an important role in prediction of success rate of ART.

**Objective:** This study was performed to evaluate whether there was any difference in follicular fluid PAPP-A, fertilization, and embryo quality between GnRH agonist long protocol and flexible GnRH antagonist multiple-dose protocol in ART cycles.

**Materials and Methods:** A total of 100 women who were candidates for ART were enrolled the study and were divided into two groups, GnRH agonist (GnRHa) long protocol (n=51) and flexible GnRH antagonist (GnRHant) multiple-dose protocol (n=49). Follicular fluid sample was obtained from a single mature follicle and follicular fluid PAPP-A level, fertilization and embryo quality of the same oocyte were evaluated in both groups.

**Results:** There was no significant difference in the mean levels of follicular fluid PAPP-A between the GnRHa protocol and GnRHant protocol (3.5±1.4 vs. 3.8±1.9, respectively). The mean levels of follicular fluid PAPP-A in fertilized oocyte and good quality embryo were comparable in GnRHa and GnRHant protocols.

**Conclusion:** Our data indicated that no differences of follicular fluid PAPP-A levels were observed between cycles using GnRHa long protocol and those of using flexible GnRHant multiple-dose protocol.

## Introduction

Ovarian follicular development is an essential factor for success rate of assisted reproductive technology (ART). Determination of fertilized oocyte and embryo quality are one of the most important purposes in ART cycles (1-4).

In recent years, different ovarian stimulation protocols have been used to increase fertilized oocyte and good quality embryos. GnRH agonists (GnRHa) and GnRH antagonists (GnRHant) are two controlled ovarian stimulation (COS) methods that have widely used for in vitro fertilization (IVF) and embryo transfer (ET) cycles (2, 5-7). The efficacy of GnRHa and GnRHant has not been fully clarified. Most published studies, comparing GnRH agonist and antagonist protocols, have shown equivalent outcomes or only slightly worse outcomes with antagonist protocol. The quality and number of follicles are known to be improved by GnRH agonist (1, 2, 8-10).

Follicular fluid (FF) provides an important microenvironment for development of oocyte and some biochemical characteristics of the follicular fluid surrounding the oocyte may play a critical role in determining the oocyte quality, the subsequent fertilization and embryo development. Thus follicular fluid content may play an important role in prediction of success rate of ART (3, 5, 11-13). GnRHant may change the intrafollicular microenvironment and may disrupt autocrine or paracrine signaling of GnRH in ovarian cells and GnRHant can affect folliculogenesis, implantation and embryo development by a direct inhibition of IGF system. So GnRHant, in these mechanisms can be account for differences in the ART outcome compared to GnRHa (2, 14-16). Many components are involved in the developing of ovarian follicles and recently, much attention has been drawn on pregnancy-associated plasma protein-A (PAPP-A) (1, 17). 

PAPP-A is a metalloproteinase that has been identified as an IGFBP-4 protease and likely an important regulator of IGF bioavailability. It degrades inhibitory IGFBP and increase free IGF and E_2_, thus indirectly enhances FSH action on the ovary (1, 2, 17-20).Some researchers believe that PAPP-A may have a critical effect on FSH-induced folliculogenesis and may a potential marker reflecting a positive role on the follicular environment and any change in this component could potentially affect follicular development and ART outcome (1, 2, 18, 21-23).

A clear correlation between specific FF biochemical characteristics, such as PAPP-A, and oocyte quality and embryo development has not been established between different COS protocols and there are few comparative reports regarding to the FF level of PAPP-A between GnRHa and GnRHant protocols in ART cycles (1, 3, 11, 24).

The aim of this study was to evaluate whether different COS protocols such as GnRHa and GnRHant have any effect on the follicular fluid PAPP-A, and fertilization and embryo quality in ART cycles.

## Materials and methods

A total of 100 women who were candidates for ART were participated in this cross sectional study from February 2010 to March 2011. The study was approved by ethics committee of Research and Clinical Center for Infertility, Yazd University of Medical Sciences. Informed consents were obtained from all patients. The women with age <35 years old, BMI ≤25 Kg/m^2^, basal FSH <10 IU/ML and regular menstruation were enrolled the study. 

The exclusion criteria were PCOS, IVF failure ≥3 in previous ART cycles, other endocrine disorders, endometriosis and severe male factor (azoospermia or normal morphology lower than 4%). Patients were divided into two groups, GnRHa long protocol and flexible GnRHant multiple-dose protocol.

**COS protocols**

In GnRHa long protocol (n=51), pituitary down-regulation was done with SC injection of 0.5 mg/day buserelin (Suprefact, Aventis, Frankfurt, Germany) which was started on day 21 of the previous menstrual cycle. After confirmation of pituitary suppression, using serum E_2_ ≤50 pg/ml and the absence of ovarian cyst by using transvaginal ultrasound, the buserelin dose was reduced to 0.25 mg/day and was continued until the day of HCG injection. Ovarian stimulation was initiated with HMG (Menogon, Ferring pharmaceuticals, Germany) 150 IU/day on the day 2 of menstrual cycle.

In flexible GnRHant multiple-dose protocol (n=49), ovarian stimulation was performed by administration of HMG 150 IU/day on the day 2 of menstrual cycle without previous oral contraceptive pretreatment and when at least one follicle by mean diameter ≥14 mm diagnosis, 0.25 mg GnRH anagonist (Ganirelex, Organon, the Netherlands) SC daily was started and was continued until the day of HCG injection. 

In both groups, ovarian response was monitored by both transvaginal ultrasound and serum E_2_ levels and gonadotropin dosage was adjusted according to individual's response and was continued until the day of HCG injection and when at least two follicles reached a mean diameter of 18 mm, 10000 IU HCG (Pregnyl, Organon, OSS, the Netherlands) was applied IM and oocyte retrieval was performed 34-36 hours after HCG injection.

**Collection of follicular fluid**

Single follicle with a diameter greater than 17 mm was obtained individually during oocyte retrieval and contaminated follicular fluid with blood was excluded the study. Immediately after single follicular aspiration, the cumulus oocyte complex was separated from FF and then FF was centrifuged for 15 min and centrifuged FF was stored at -80°c until the samples were all completed. Also the obtained single oocyte was cultured in a separated culture dish and ICSI was performed on that oocyte 4-6 hours after oocyte retrieval. 

Fertilization of the same oocyte was evaluated 16-18 hours after ICSI and fertilized oocyte was defined as zygote with two pronucleous (2PN). Quality of embryo was assessed on fertilized oocyte 48 hours after ICSI and was scored based on the shape, number and fragmentation of blastomers (25) and embryo score ≥18 was decided to indicate good quality in our center. PAPP-A levels in FF were determined using enzyme-linked immunosurbent assay (ELISA) kit (LDN Labor Diagnostika Nord, GmbH & Co.KG)

**Statistical analysis**

The parameters were analyzed with the SPSS version 15.0 (SPSS Inc., Chicago, IL). The results were compared between the two groups and statistically analyzed using the Student’s *t*-test or chi-square test where appropriate. P-value less than 0.05 was considered to be statistically significant.

## Results

There was no significant difference between two protocols in regard to basic characteristics' of patients (Table I). Etiologies of infertility were similar in two protocols (Table II). There was no significant difference in the mean levels of follicular fluid PAPP-A between the GnRHa long protocol and GnRHant protocol (3.5±1.4 versus 3.8±1.9, respectively). The mean levels of follicular fluid PAPP-A in fertilized oocyte were similar in two protocols (3.5±1.6 in GnRHa versus 4.2±2.1 in GnRHant) (Table III). 

The mean levels of follicular fluid PAPP-A with good quality embryo were comparable in GnRHa and GnRHant protocols (3.6±1.6 versus 4.5±1.6, respectively) (Table IV). There were no significant correlation between follicular fluid PAPP-A levels, and fertilization and good quality embryo in GnRHa and GnRHant protocols. 

**Table I:** Basic characteristics of patients in two protocols

**Variable** **s**	**GnRHa**	**GnRHant**	**p-value**
Age (year)	28.8 ± 4.2	30.4 ± 3.9	0.062
Infertility duration (year)	7.2 ± 4.1	8.7 ± 4.7	0.122
BMI (kg/m^2^)	22.2 ± 2.4	23.6 ± 1.3	0.059
Basal FSH (mIU/ml)	6.1 ± 2.2	6.9 ± 1.7	0.095

**Table II:** Infertility etiology in two protocols

**Variable** **s**	**GnRHa ** **n (%)**	**GnRHant** **n (%)**	**p-value**
Male factor	22 (43.1%)	15 (30.65%)	
Ovarian factor	6 (11.8%)	7 (14.3%)	
Tubal factor	10 (19.6%)	6 (12.2%)	
Unexplained	8 (15.7%)	9 (18.4%)	
Mixed	5 (9.8%)	12 (24.5%)	
Total	51 (100%)	49 (100%)	0.067

**Table III:** Mean level of follicular fluid PAPP-A in fertilized oocyte between two protocols.

**Variable **	**Fertilization**		**p-value**
	**GnRHa**	**GnRHant**	
Follicular fluid PAPP-A levels (µg/ml)	3.5 ± 1.6	4.2 ± 2.1	0.612

**Table IV:** Mean level of follicular fluid PAPP-A in good quality embryo between two protocols

**Variabl **	**Good quality embryo**		**p-value**
	**GnRHa**	**GnRHant**	
Follicular fluid PAPP-A levels (µg/ml)	3.6 ± 1.6	4.5 ± 1.6	0.643

## Discussion

Overall, a clear correspondence between specific follicular fluid biochemical characteristics, and fertilization and embryo development has not been established in the literature. It appears that ovarian folliculogenesis requires dynamic interactions between mature oocytes, granulose cells and regulatory factors present in the follicular fluid and suitable follicular environment is necessary for good oocyte quality (3, 5).

In the present study, the mean levels of follicular fluid PAPP-A were similar in GnRHa long protocol and flexible GnRHant multiple-dose protocol (3.5±1.4 versus 3.8±1.9, respectively) and we found any correlation between FF levels of PAPP-A, and fertilization and good quality embryo in GnRHa protocol compared to those of GnRHant protocol.

Tzu-Hao *et al* (24) evaluated IGF-II, IGFBP-3, IGFBP-4, and PAPP-A levels in FF. They found high levels of IGF-II, IGFBP-3 and IGFPB-4, and low levels of PAPP-A in FF at the time of oocyte retrieval. They suggested that low FF concentrations of PAPP-A and high levels of IGF-II, IGFBP-3, and IGFBP-4 in ovarian follicles correlated with better oocyte maturation and early embryo development and these components may be used for predicting which oocytes would be successfully fertilized and developed into early embryo. Based on our results, we cannot used FF levels of PAPP-A as a predictor of oocyte fertilization and good quality embryo. 

Rezabek *et al* found any correlation between PAPP-A follicular fluid concentrations in women undergoing IVF with OHSS risk and they concluded pathological response in hormonal stimulation leading to OHSS was not correlated to the follicular fluid levels of PAPP-A (26).

In similar study, Stanger *et al* showed that at PAPP-A levels more than 200 µg/l, there was no correlation noted with the ability of the oocyte fertilization or cleavage. They found no significant difference in the mean levels of PAPP-A for the two stimulation protocols, clomiphene citrate alone or in combination with human menopause gonadotropin, and it seems PAPP-A levels may provide an index of follicle maturity but not of the pregnancy potential of the ovum (27).

Despite many advantages on the clinical results of COH, the role of GnRHa and GnRHant protocols on folliculogenesis and its effect on intrafollicular microenvironment remains controversial (1, 28-30). Jihyun *et al* evaluated the different doses of GnRH agonist on intrafollicular PAPP-A levels in COH cycles. They concluded that the dose of GnRHa may have a significant effect on the intrafollicular environment, reflected by the expression of PAPP-A (1). Moos *et al* evaluated follicular fluid and serum levels of PAPP-A in patients undergoing IVF. According to their results, they suggested that a considerable amount of PAPP-A is accumulated in the ovarian follicles of women undergoing IVF (19.775 IU median), but intrafollicular PAPP-A dose not substantially elevate the PAPP-A serum concentration and it seems PAPP-A acts only locally and cleaves IGFBP-4 within the ovarian follicle (20).

IGFs are stimulating steroidgenesis and IGFBPs are effective antigonadotropins. Both of them play an essential role on follicular growth. IGFBP-4 is an inhibitor of IGF activity and FSH stimulated granulose steroidgenesis and PAPP-A, by cleaving IGFBP-4, is providing higher IGF-II activity in the follicles (5, 31-33).

There are few studies in regard to follicular fluid PAPP-A between the two protocols, GnRHa and GnRHant protocols (1-2, 11, 24). Young Sik *et al* showed that the mean levels of follicular fluid PAPP-A in GnRHa long protocol was 0.53±0.25 mIU/ml compared to 0.50±0.31 mIU/ml in GnRHant protocol (2). Similar to our results, no significant differences were noted between the concentration of FF PAPP-A in GnRHa and GnRHant protocols. They observed that there were significant differences in the IGF-II, IGFBP-4 levels between two groups. The effect of higher IGF-II concentration appeared to be inhibited by higher IGFBP-4 in the GnRHa long protocol compared to the GnRHant protocol and they concluded steroidogenesis of dominant follicle in cycles using GnRHant was not different from that in cycles using GnRHa (2). Their findings are in agreement with our results for follicular fluid PAPP-A levels between two protocols.

In present study, we evaluated FF concentration of PAPP-A, while there are several endocrine/paracrine and ovarian local regulatory factors that may be have positive or negative effects in IVF-ET cycles. Although remarkable progress has been made in understanding the biology of PAPP-A during recent years, further studies is needed to evaluate other underlying mechanisms or factors that are involved in fertilization and embryo development in IVF-ET cycles. 

## Conclusion

Our data indicated that no differences of follicular fluid PAPP-A levels were observed between cycles using GnRHa long protocol and those of using flexible GnRHant multiple-dose protocol and it appears follicular fluid PAPP-A cannot be considered as a marker of fertilization and embryo quality in IVF-ET cycles that are used GnRHa or GnRHant protocols.
